# Effect of Process Conditions and Colloidal Properties of Cellulose Nanocrystals Suspensions on the Production of Hydrogel Beads

**DOI:** 10.3390/molecules26092552

**Published:** 2021-04-27

**Authors:** Nicola Ferrari, Cecilia Ada Maestri, Paolo Bettotti, Mario Grassi, Michela Abrami, Marina Scarpa

**Affiliations:** 1 Department of Physics, University of Trento, 38123 Trento, Italy; ciproferro@gmail.com (N.F.); maestri.cecilia@gmail.com (C.A.M.); paolo.bettotti@unitn.it (P.B.); 2 Department of Engineering and Architecture, University of Trieste, 34127 Trieste, Italy; mario.grassi@units.it (M.G.); michela.abrami@units.it (M.A.); 3 Istituto Nazionale Fisica Nucleare (INFN), 38123 Trento, Italy

**Keywords:** cellulose nanocrystals, colloidal fluid dynamics, hydrogels, extrusion dripping, centrifuge-driven dripping

## Abstract

The influence of the physical, rheological, and process parameters on the cellulose nanocrystal (CNC) drops before and after external gelation in a CaCl_2_ solution was investigated. The dominant role of the CNC’s colloidal suspension properties, such as the viscous force, inertial, and surface tension forces in the fluid dynamics was quantitatively evaluated in the formation of drops and jellified beads. The similarity and difference between the behavior of carbohydrate polymers and rod-like crystallites such as CNC were enlightened. Pump-driven and centrifugally-driven external gelation approaches were followed to obtain CNC hydrogel beads with tunable size and regular shape. A superior morphological control—that is, a more regular shape and smaller dimension of the beads—were obtained by centrifugal force-driven gelation. These results suggest that even by using a simple set-up and a low-speed centrifuge device, the extrusion of a colloidal solution through a small nozzle under a centrifugal field is an efficient approach for the production of CNC hydrogel beads with good reproducibility, control over the bead morphology and size monodispersion.

## 1. Introduction

The microscale manipulation of colloidal macromolecule suspensions is of high significance in view of the use of microfluidics and 3D ink-jet printing to obtain hydrogels with superior properties and on-demand functionalities [1,2]. These hydrogels find applications in analytical, biotechnology, and medical field as drug delivery systems [3], cell scaffolds [4], tissue engineering supports [5], and matrices for biosensors [6]. Moreover, regularly sized gel beads are receiving interest as possible components of liquid system displays or other sophisticated futuristic materials [7]. The extrusion dripping of droplets of a colloidal suspension through a microfluidic system followed by external gelation is a well-known method to produce hydrogel beads. Various approaches are utilized to extrude the colloidal suspensions through a terminal nozzle, such as the force of gravity or the centrifugal force; then, the droplets quickly become gels in contact with an appropriate solution. Hydrogel objects with good uniformity, tunable size, shape, and composition have been obtained by this approach, in particular using alginate, which is a representative carbohydrate polymer and Ca^2+^ as a gelation agent [8]. The factors affecting the bead formation such as the experimental setup, the fluid and the gelling solution characteristics, e.g., tip size, collecting distance, viscosity, or surface tension, have been extensively studied and reviewed [8,9]. According to these studies, the colloidal suspension properties play a dominant role in the fluid dynamics at the microscale. A peculiar carbohydrate colloidal system which came recently into play is nano-sized cellulose, in the form of nanofibers (CNF) and nanocrystals (CNC) as hydrogel building blocks [10]. Although CNCs are rigid, rod-like crystalline nano-objects, they form 3D interconnected hydrogel networks [11], and beads or core–shell capsules are obtained by the external or inverse gelation of CNC, respectively [12]. The morphology, rheology, and surface properties of CNCs obtained with various procedures have been extensively studied [13]. Conversely, scarce information is available on the dynamics of the CNC suspensions and on the synthesis of CNC hydrogels under external forces. The lack of this information limits the progress toward a rational design of operation protocols for CNC hydrogel production. In particular, microfluidic systems operating under centrifugal forces have never been applied for the CNC hydrogel manufacturing. This simple approach offers several advantages, such as reproducibility, control of bead size and shape, and production yields. Herein, we investigated the role of the fluid dynamics of the CNC suspensions and of the processing parameters in the hydrogel bead production by pump and centrifuge-driven external gelation. A precise description of the size and shape of the beads obtained under various conditions is reported, and the potential of the centrifugally-driven microfluidic systems is proven.

## 2. Results

### 2.1. Physical–Chemical Peroperties of CNC Solutions

**Figure 1:**
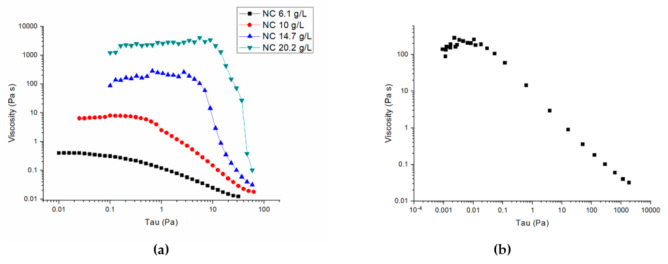
(**a**) Viscosity versus stress curves of CNC solutions. (**b**) Flow curve of the 14.7 gL^−1^ CNC solution The measurements were performed at 25 °C. The flow curve clearly demonstrates the shear thinning behavior of the material.

Rheological measurements in the concentration range used in the present work (i.e., 6.1–20.2 gL^−1^) showed that all the investigated samples behaved as liquids, since the viscous modulus G″ was higher than the elastic modulus G′ (data and details of the rheological characterization are reported in the Supplementary Material, Appendix A and Figure S1). The viscosity values at variable applied stress are shown in Figure 1a, and the representative flow curve of the 14.7 gL^−1^ CNC solution is shown in Figure 1b. These curves confirm that CNC solutions are shear-thinning pseudo-plastic fluids [14]. Moreover, viscosity increases as CNC concentration increases in the range tested.

### 2.2. Production of Hydrogel Beads by Pump-Driven External Gelation

**Figure 2:**
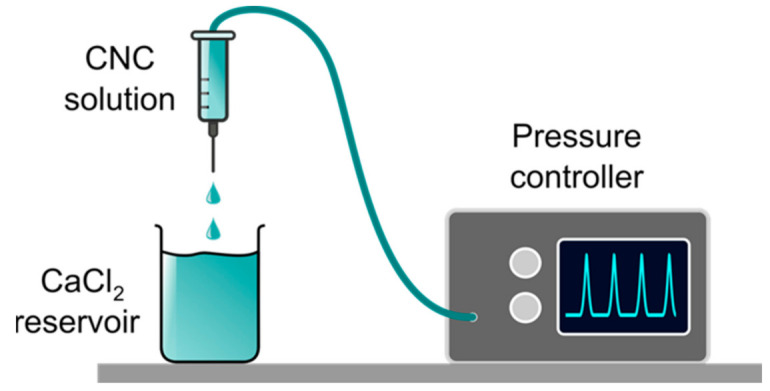
Sketch of the set-up used to generate the CNC.

Figure 2 reports a sketch of the set-up used to produce the hydrogel beads by syringe dripping of CNC solutions in an external gelation CaCl_2_ bath. To deliver the CNC solution through the extrusion nozzle, a controlled and constant pressure (Δ*P*) was applied. For a series of experiments, Δ*P* were kept in the range 8–13 kPa, depending on the CNC concentration, in order to obtain a flow rate $Q=3.0{\;\times \;10}^{\;-3}{\mathrm{mLs}}^{-1}$. At these Δ*P* values, we did not observe the formation of drop satellites. On the other hand, a clear dripping to jetting transition was observed if ΔP was increased to 90–95 kPa, that is, when the flow rate reaches a critical value $Q_{j}∼7.0{\;\times \;10}^{-2}{\;\mathrm{mLs}}^{-1}$ using a 14.1 gL^−1^ CNC solution.

**Figure 3:**
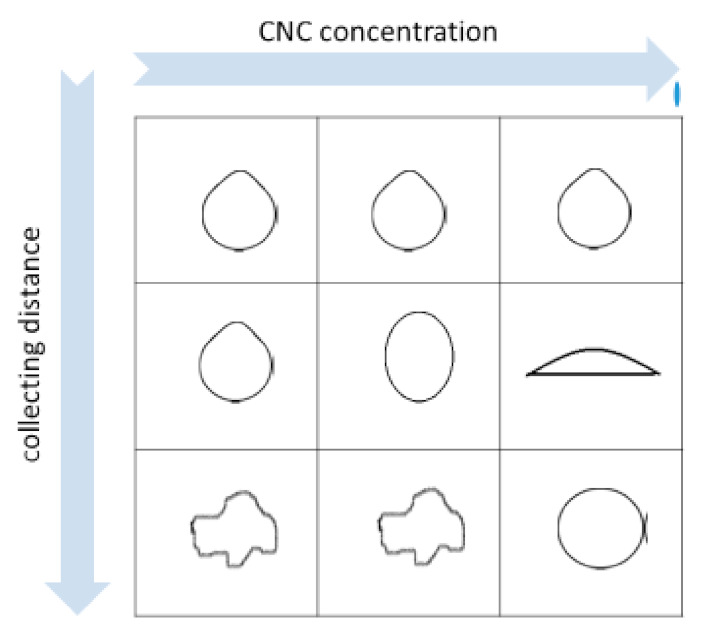
Sketch of the bead shape at increasing CNC concentration and collecting distance.

An aqueous solution containing 0.1 M CaCl_2_ was used as gelation bath. This CaCl_2_ concentration value was found as the optimal one, since it allows fast and stable gelation of CNC. Higher salt concentrations were not considered in order to avoid substantial perturbation of the chemical and physical properties of the aqueous bath. Moreover, the gelation process is diffusion controlled [12], and higher Ca^2+^ concentrations are expected not to leave a relaxation time long enough for the droplets to regain their spherical shape after colliding with the gelling bath. In some experiments, 60 μM Tween 20 was added to lower the surface tension. Representative pictures of the obtained beads are shown in Appendix B of the Supplementary Material, Figure S2. The morphology of the hydrogel beads obtained by changing (a) the concentration of the CNC solution, (b) the collecting distance h, and (c) the surface tension σ of the gelling bath is described in the Supplementary Material, Table S1. All the other experimental parameters were kept constant. A sketch summarizing the influence of the two more effective parameters (i.e., collecting distance and CNC concentration) on the bead shape is shown in Figure 3.

The largest dimension (*d_max_*) of the beads obtained by the syringe apparatus of Figure 2 was always in the range 2–4 mm. A deformation in bead’s shape occurred at low viscosities and high collecting distance (see Table S1 and Figure 3), and it was probably due to the drag forces of the CaCl_2_ solution and to the impact of the droplet with the gelling bath surface. In fact, the beads obtained with higher CNC concentrations (i.e., CNC solution viscosity η > 5 Pa⋅s at τ=10 Pa) are more uniform and regular in shape. The beads become more flat and concave when Tween20 lowers the surface tension of the gelling bath, producing a spreading effect of the CNC drop. The beads obtained at 14.7 gL^−1^ CNC concentration have a more regular morphology, and we measured the minimum (*d_min_*) and maximum (*d_max_*) dimension (details in the Methods) to calculate the two different dimensionless shape indicators—that is, the sphericity factor (*SF*) and the aspect ratio (*AR*)—at different collecting distances. These values are reported in Table 1. In particular, *SF* values always > 0.05 indicate that the beads are spherical ellipsoids at all the collecting distances, but in spite of the large variability of the averaged *SF* value, some spherical beads were observed at 20 cm collecting distance.

Under the conditions of low flow rate of the CNC solution in the syringe needle, the diameter of the droplets was not affected by the flow rate itself (experimental data not shown in the present work, which are in accord with those reported in [15]). In this case, the bead diameter ($d_{p}$) can be predicted by the Tate’s law modified to consider the bead shrinkage associated with the gelation process and the volume of the residual solution remaining at the dripping tip after droplet detachment, Equation (1):

(1) $$d_{p}{=k}_{LF}k_{SF}\;{\left(\frac{0.006d_{t}\sigma }{\rho g}\right)}^{1/3}$$

where $d_{t}$ = 636 ± 4 μm is the outer diameter of the syringe tip, $k_{\mathrm{SF}}=\frac{d_{p}}{d_{d}}\;$ is the shrinkage factor*,* that is the ratio between the drop diameter after ($d_{p}$) and before ($d_{d}$) the gelation process. $k_{\mathrm{LF}\;}={\left(\frac{V_{\mathrm{real}}}{V_{\mathrm{ideal}}}\right)}^{1/3}$ is the liquid lost factor, where $V_{\mathrm{ideal}}$ is the drop volume according to the theoretical Tate’s law [8,16], Equation (2):

(2) $${\rho \mathrm{gV}}_{\mathrm{ideal}}{=2\pi r}_{t}\sigma$$

and ρ=1.005 kgdm^−3^ is the density we measured for the 14.7 gL^−1^ CNC solution (details in Section 3.2), $g=9.813{\;\mathrm{ms}}^{-2}$ is the gravitational acceleration ${r\;}_{t}=636\;\mu m$, is the external radius of the capillary, and ${\;\sigma =72\;\mathrm{mNm}}^{-1},$ [17,18]) is the surface tension. $V_{real}$ is the experimental volume of the beads.

A $V_{ideal}\;$ value of $\;(1.46\;\pm \;0.03{)\;\times 10}^{-2}\;\mathrm{mL}\;$ was calculated from the theoretical Tate’s law and a $V_{real}$ value of $(1.08\;\pm \;0.02{)\;\times 10}^{-2}\;\mathrm{mL}$ was found (details in the Supplementary Material, Appendix C). Therefore, a value of $k_{LF}=0.90\pm 0.01$ was obtained.

A shrinkage factor $k_{SF}=1.10\;\pm \;0.01\;$ of the Ca^2+^ crosslinked CNC beads was calculated from the ratio ${\left(\frac{V_{p}}{V_{d}}\right)}^{1/3}$, where $V_{d\;}{=V}_{\mathrm{real}}.\;$ The experimental value of $V_{p}=\;\left(1.19\;\pm \;0.07\right){\;\times \;10}^{-2}\;\mathrm{mL}\;$ was obtained as described in the Supplementary Material, Appendix C. The $k_{SF}$ value indicates that differently from alginates [8], CNC drops do not shrink upon gelation; conversely, a slight volume expansion is observed. Since this expansion is associated with the gelation process, the diffusion of some CNCs outside the drop, before a compact gel shell is formed, could contribute to this effect.

### 2.3. Determination of the Ohnesorge Number

The non-dimensional Ohnesorge number (*Oh*) was used to obtain a quantitative evaluation of the importance of the viscous force to surface tension force in determining the bead formation. In particular, under our experimental conditions, the *Oh* number could account for the effect of the CNC solution properties on bead shape transition observed at different collecting distances [8]. This number is defined as Equation (3):

(3) $$\mathrm{Oh}=\frac{\eta }{{\left({\rho d}_{d}\sigma \right)}^{1/2}}$$

where η (Pa s) is the dynamic viscosity and is a function of shear rate $\dot{\gamma \;}$ as shown in Figure 1b.

Fitting the data of Figure 1b to the equation that holds for polymer solutions [19], Equation (4):

(4) $${\eta =\eta }_{0}{\dot{\gamma \;}}^{n-1}$$

where $\eta_{0}$ is the viscosity at zero shear strain rate and the exponent *n* depends on the degree of thinning, we obtain $\eta_{0}{=13\;\pm \;2\;\mathrm{Pas}}^{-1}$ and n=0.40 ± 0.03.

From the equation of the maximum value $\left|{\dot{\;\gamma }}_{\max}\right|$ of the shear rate in a pipe [20], Equation (5):

(5) $$\left|{\dot{\;\gamma }}_{\max}\right|=\left(\frac{3n+1}{n}\right)\times \left(\frac{Q}{{\pi r}_{t}}\right)$$

and on the basis of the *n* value and the parameters of the set of experiments performed with a solution containing 14.7 gL^−1^ CNC, i.e., $r_{t}=225\;\pm \;5\;\mu m$ and $Q=\left(3.0\;\pm \;0.2\right){\times 10}^{-9\;}m^{3}s^{-1}$, we obtain $\left|{\dot{\;\gamma }}_{\max}\right|{=500\;\pm \;20\;\mathrm{rads}}^{-1}$ which, according to Figure 1b, gives η=0.06 ± 0.01 Pas.

Since $d_{d}=(2.83\;\pm \;0.04{)\;\times \;10}^{-3}m\;$(as calculated from the volume of the detaching droplet, details reported in the Supplementary Material, Appendix C), the *Oh* value at CNC concentration of 14.7 gL^−1^ is 0.13, which is lower than the threshold value of 0.24 above which the spherical shape of the beads of carbohydrate polymer solutions has been reported [8].

### 2.4. Production of Beads by a Centrifugal-Force-Driven Micronozzle System External Gelation

**Figure 4:**
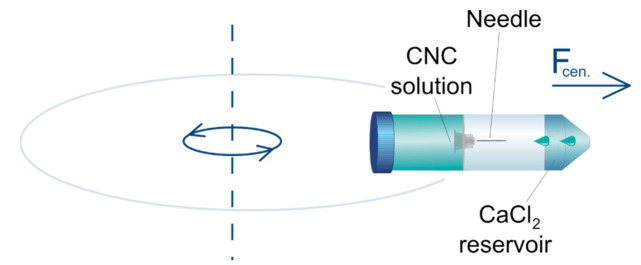
Sketch of the rotating micro-nozzle set-up.

We investigated the production of the hydrogel beads under the artificial gravity (*g*) by using a home made centrifugal-force-driven micronozzle system, the sketch of which is shown in Figure 4.

In this case, the aqueous solution containing 14.7 gL^−1^ CNC flows from a micro-sized tip and impacts into a receiving tube containing 0.1 M CaCl_2_ solution, which is held in a flying bucket of a centrifuge rotor. Representative bead images obtained by this procedure are shown in Figure S3 (Supplementary Material, Appendix D). It should be noticed that the beads were characterized by a rather regular shape with a size dispersion of about ±5%. We did not notice the presence of hydrogel filaments and pearl-necklace beads. Moreover, the size distribution of the beads was peaked at a single value. These data suggest that the experiments were performed under the dripping without satellites regime. To evaluate the flow regime inside the capillary, we calculated the Reynolds number, equation (6).

(6) Re=vdtρη

**Figure 5:**
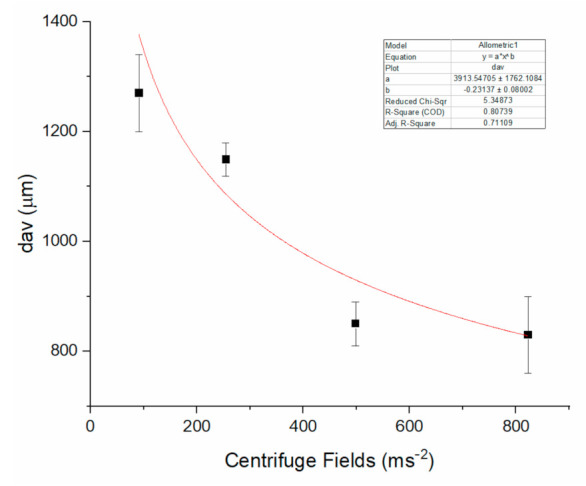
Average bead size ($d_{\mathrm{av}})$ versus centrifugal acceleration, obtained by 14.7 mgmL^−1^ CNC solution.

Considering an average solution viscosity at high shear rates, *η*, of about 100 Pa s, a mean velocity of fluid ${v=291\;\mathrm{cm}\;s}^{-1}$, and $d_{t\;}=1.24{\;\times 10}^{-3}\;\mathrm{cm}$, an Re value of about ${4\;\times \;10}^{-2}$ was obtained, indicating that the CNC suspension was in the inertial laminar flow regime. The bead size was only slightly dependent on the nozzle internal diameter, as observed by varying this parameter in the range 610–1455 µm. Conversely, the collection distance affected both the shape and the dimensions of the hydrogel beads. In particular, the bead size was measured by varying the collection distance, keeping constant the other experimental settings, such as the diameter and length of the capillary tip, which were $d_{t}=1240\;\pm \;10\;µm\;\mathrm{and}\;l=22\;\mathrm{mm},\;$ respectively, and the centrifuge field, which was set at 9.3×g. The average values of $d_{\max}\;$ and $d_{\min}$ of a sample of 20 drops together with the SF and AR factors are reported in Table 2 and show that the beads increased in size and become more elliptic at increasing the collection distance between the end of the capillary and the gelling solution. Moreover, centrifugal acceleration dictates the size and the SF and AR factors, according to the data of Table 3 and Figure 5 where the diameters ($d_{\max},$ $d_{\min})$, their mean value ($d_{\mathrm{av}})$ together with the morphology parameters obtained at centrifugal acceleration varied in the interval (9.3–84.0)× g  are reported.

To obtain a rational picture of the size dependence on the experimental parameters, we consider that the droplet detaches from the nozzle when the gravitational pseudo-force surpasses the surface tension force [16], Equation (7):

(7) $${m\omega }^{2}{r=\pi d}_{t}\sigma$$

where ω is the angular frequency and *r* is the distance from the center of rotation, from which a theoretic bead diameter can be calculated, Equation (8):

(8) $$d_{p}={\left(\frac{0.006d_{t}\sigma }{{\rho \omega }^{2}r}\right)}^{1/3}$$

Analogously to the equations obtained for the production of beads by extrusion dripping, the bead shrinkage and the liquid lost factors should be taken into account. However, in light of the small contribution of these factors in the dripping experiments, in the following analysis, we neglect them. If the simplified calculation in Equation (8) holds, we estimate a theoretical $d_{p}\;$ value of the order of 1800 µm for a solution of 14.7 gL^−1^ CNC at $d_{t}=1240\;\pm \;10\;\mu m\;$ and $\omega^{2}r=91.4{\;\mathrm{ms}}^{-2}$. Equation (8) justifies the small change of $d_{p}$ we observed by changing the nozzle tip diameter by a factor of 2.4. Moreover, the best fit for the experimental $d_{p}$ values versus the centrifuge acceleration reported in Figure 5 provides the function $d_{p}=a{\left(\omega^{2}r\right)}^{b}\;$ where a is a constant, and the exponent b=−(0.23 ± 0.08) is close to the value of –0.33 expected from Equation (8). The theoretical $d_{p}$ value of about 1800 μm  is close to the experimental average size we measured at a collection distance of 15 mm (i.e., $d_{\mathrm{average}}=1850\pm \;80\;\mu m$). Conversely, shorter collecting distances produce more spherical beads with sizes smaller than the theoretical one. For what concerns the bead shape, a similar behavior has been reported for the alginate beads and ascribed to the variation of kinetic energy (and drop velocity) at the impact [21]. Our data indicate also that the size increases with the collecting distance, and this effect could be attributed to the fluid expansion upon ejection from the capillary nozzle. The increase of diameter of viscoelastic fluids ejected into air from a capillary nozzle is a complex phenomenon that is mainly dependent on fluid relaxing after the normal stress developed in the capillary [22]. Middleman and Gavis reported an expansion of about 20% for a 2% carboxymethyl cellulose suspension ejected into air at a velocity of about 20 ms^−1^, which is comparable to that reached in the centrifuge bucket. It can also be expected that the jet expansion is more pronounced at the very low Reynolds number estimated in our conditions, since relatively low velocity and high viscosity contribute to viscoelastic response of the fluid to capillary stress and relaxation [23].

## 3. Materials and Methods

### 3.1. Materials

All the reagents were from Sigma Aldrich-Merck (Milan, Italy). Cellulose pulp was a kind gift of Sca (Sundsvall, Sweden). First, 10 g of pulp were swollen in water for 1 h and bath sonicated for 30 min. Then, 160 mg TEMPO, 1 g NaBr, and 35 mL 12% NaClO were added according to the method of Saito and Isogai [24], which was slightly modified as described in a previous report [25] to introduce carboxylic functions. At the end of the reaction, the slurry was tip sonicated, and a transparent suspension of CNCs was obtained. The presence of the carboxylic groups was assessed on the basis of the FTIR spectrum, which showed a sharp peak at around 1610 cm^−1^. The details of the nanomaterial characterization are reported in Appendix E of the Supplementary Material. The final concentration was reached by using a Rotorvapor (Buchi, Essen, Germany). Rheological measurements were performed by a stress controlled rotational rheometer (Haake Mars Rheometry, 379–0200 Thermo Electron GmbH, Karlsruhe, Germany) equipped by parallel plate geometry.

### 3.2. Measurement of the Density of CNC Solutions

The density (ρ) of a CNC solution CNC was measured at 20 °C using a pycnometer, the volume of which was calculated by measuring the mass of the deionized water necessary to fill it and taking a water density value of 0.9982 KgL^−1^ at 20 °C. Then, the density of a 14.7 gL^−1^ CNC solution was calculated using this volume and the experimental value of the mass of the CNC solution, finding 1.0050 ± 0.0003 kgL^−1^ (N = 7).

### 3.3. Extrusion Dripping Experiments

To produce CNC beads by extrusion dripping and external gelation in a CaCl_2_ bath, we used a set-up consisting in a syringe equipped with a metallic needle with an outer diameter (d_t_) and an inner diameter (d_i_) of 636 ± 4 μm and 225 μm, respectively, connected to Elveflow Microfluidics Flow/Pressure Control System (Elvesys, Paris, France). A CaCl_2_ reservoir was located at a variable distance from the needle tip.

### 3.4. Centrifugally Driven Pulse-Free Flow Experiments

CNC beads were produced by a centrifugally driven pulse-free flow approach consisting of a pipette tip working as a CNC solution reservoir. This pipette tip was sealed by an adhesive to a glass or polyethylene capillary of variable length from which droplets of the CNC solution were released. This device was held firmly in a fixed position by inserting it in the central hole of a Teflon support. Then, the Teflon support was fit inside a 50 mL Falcon tube, the bottom of which contained the gelling bath; i.e., a 0.1 M CaCl_2_ solution. The Falcon tube was placed into holes of a swinging bucket rotor of a centrifuge (Thermo SCIENTIFIC SL16R, ENCO, Venezia, Italy). Centrifugal accelerations in the range  (9.3–84.0)× g  produced the pseudo force, which drove the buckets in a horizontal position and induced the fluid flow from the reservoir to the gelling bath.

### 3.5. Bead Analysis

The size and shape of the beads were investigated using the experimental photos and the image processing platform ImageJ (National Institutes of Health, USA) [26].

The beads produced by pump-driven external gelation were withdrawn from the gelling bath, and the excess of solution was gently removed with a blotting paper. Then, the beads were laid down on a support covered with graph paper, and photographs were taken.

The beads produced by a centrifugal-force-driven micronozzle system were poured into a Petri dish containing a 0.1 M CaCl_2_ solution, and the photos were taken by a stereo microscope (LEICA MZ16FA, Milano, Italy).

The maximum diameter ${(d}_{\max})$ and the minimum diameter ($d_{\min})$ defined as the diameter perpendicular to the maximum one were measured. The bead shape was described by the sphericity factor (*SF*) and the aspect ratio (*AR*), see Equations (9) and (10):

(9) $$\mathrm{SF}=\frac{d_{\max}-d_{\min}}{d_{\max}{+d}_{\min}}$$

(10) $$\mathrm{AR}=\frac{d_{\max}}{d_{\min}}$$

### 3.6. Shrinkage Factor (SF) Determination

The shrinkage factor, Equation (11):

(11) $$k_{\mathrm{SF}\;}=\frac{d_{p}}{d_{d}}$$

where $d_{p}\;\mathrm{and}\;d_{d}$ are the bead diameter after and before gelation respectively, was determined from the volume of the gelled bead and of the CNC solution drop, respectively.

The volume of the solution drops was calculated from the experimental weight of the drops using the density value of the CNC solution. The volume of the gelled beads was experimentally measured. Details of the procedures used to this purpose are reported in the Supplementary Material, Appendix C.

### 3.7. Lost Factor Determination

The liquid lost factor, Equation (12):

(12) $$k_{\mathrm{LF}}={\left(\frac{V_{\mathrm{real}}}{V_{\mathrm{ideal}}}\right)}^{1/3}$$

where $V_{\mathrm{ideal}}$ is the ideal drop volume and $V_{\mathrm{real}}$ is the experimental volume, was calculated using the theoretical $V_{\mathrm{ideal}}$ value according to the Tate’s law [8] and obtaining the value of $V_{\mathrm{real}}$ from the drop weight measurement, as described in detail in the Supplementary Material, Appendix C.

## 4. Conclusions

In conclusion, in this paper, we highlighted the physical parameters and the processing conditions to control the hydrogel bead production by using a pump-driven and a centrifugal-force-driven external gelation. The latter is a superior approach to fabricate size and shape-controlled beads by using simple and cheap laboratory equipment. In fact, with the aid of centrifugal fields, smaller and round shaped beads can be obtained. Beyond the possible application of this result, our systematic investigation provides an overview on properties of CNC solutions as a fluid and estimates the role of forces (viscous, inertial, and surface tension) in the CNC solution jets. By this study, we observed important differences between the behavior of CNC solutions and the carbohydrate polymers such as alginates. In general, the CNC bead dimensions are larger than those of carbohydrate polymers, and their shape is more irregular. This is probably due to the crystallinity of CNC, which could affect both the folding at the nanoscale and the availability of carboxylate groups for Ca^2+^ coordination. Moreover, we observed that CNC solutions undergo tensile stress inside a capillary and CNC drops do not shrink upon gelation; conversely, they slightly expand.

## Figures and Tables

**Table 1:** Bead dimension and shape at variable collecting distance. CNC concentration 14.7 gL^−1^. *SF* $=\frac{d_{max}\;-\;d_{min}}{d_{max}+d_{min}}$; $AR=\;\frac{d_{max}}{d_{min}}.$ The reported values are the average values on a 20-bead sample.

Collecting Distance (cm)	*d_max_* (µm)	*d_min_* (µm)	SF	AR
1	3000 ± 100	2600 ± 100	0.07 ± 0.04	1.15 ± 0.08
2	3100 ± 200	2600 ± 100	0.09 ± 0.06	1.2 ± 0.1
3	3200 ± 200	2600 ± 200	0.10 ± 0.06	1.2 ± 0.2
5	3600 ± 100	2300 ± 200	0.22 ± 0.08	1.5 ± 0.2
20	3200 ± 200	2600 ± 200	0.10 ± 0.08	1.2 ± 0.2
30	3800 ± 200	2600 ± 200	0.20 ± 0.08	1.5 ± 0.3

**Table 2:** Dimensions and shape parameters of the beads generated by a centrifugal-force-driven system at variable collecting distances. The reported values are the average values on a 20-bead sample.

Collecting Distance (mm)	*d_max_* (µm)	*d_min_* (µm)	SF	AR
2	1270 ± 60	1180 ± 70	0.037	1.08
5	1710 ± 70	1520 ± 80	0.059	1.125
10	1930 ± 90	1570 ± 60	0.103	1.23
15	2000 ± 100	1710 ± 50	0.078	1.17

**Table 3:** Dimensions and shape parameters of beads as a function of the centrifuge field. CNC concentration 14.7 gL^−1^, collecting distance 2 mm. *SF* $=\frac{d_{max}-d_{min}}{d_{max}+d_{min}}$; $AR=\frac{d_{max}}{d_{min}}$. The reported values are the average values on a 20-bead sample.

RCF *xg*	*d_max_* (µm)	*d_min_* (µm)	SF	AR
9.3 ± 0.1	1270 ± 60	1180 ± 70	0.037	1.08
26.0 ± 0.1	1149 ± 40	1050 ± 20	0.045	1.09
50.9 ± 0.1	850 ± 30	760 ± 50	0.056	1.12
84.0 ± 0.2	830 ± 70	700 ± 70	0.085	1.19

## Data Availability

Not applicable.

## Supplementary Materials

The following are available online, Appendix A and Figure S1: Rheological characterization of CNC solutions; Appendix B and Figure S2: Production of beads by pump-driven external gelation; Table S1: Bead profiles at different experimental settings; Appendix C: Experimental drop and bead volumes and calculation of lost and shrinkage factors. Appendix D and Figure S3: Production of beads by a centrifugal-force-driven micro-nozzle system. Appendix E: Morphological characterization of CNC. Figure S4: AFM image of CNC.

## Sample Availability

Samples of the hydrogels are available from the authors.
