# Supplementary material for: Effect of Process Conditions and Colloidal Properties of Cellulose Nanocrystals Suspensions on the Production of Hydrogel Beads
Source: Molecules. 2021 Apr 27;26(9):2552. doi: 10.3390/molecules26092552 (PMC8125024; doi:10.3390/molecules26092552)
Supplement: Supplementary file 1 [file molecules-26-02552-s001.zip › molecules-1179462-supplementary.pdf]

Article

Effect of Process Conditions and Colloidal Properties of Cellulose Nanocrystals Suspensions on the Production of Hydrogel Beads

Nicola Ferrari [1], Cecilia Ada Maestri [1], Paolo Bettotti [1], Mario Grassi [2], Michela Abrami [2] and Marina Scarpa [1,3,*]

[1] Department of Physics, University of Trento, 38123 Trento, Italy
[2] Department of Engineering and Architecture, University of Trieste, 34127 Trieste, Italy
[3] Istituto Nazionale Fisica Nucleare (INFN), 38123 Trento, Italy
* Correspondence: marina.scarpa@unitn.it

Supplementary Materials

Appendix A Rheological characterization of CNC solutions

In order to determine the elastic (G'; elastic modulus) and viscous (G"; viscous modulus) characteristics of the aqueous systems containing a concentration of CNC variable in the range 6.1-11.4 gL^{-1}, stress sweep and frequency sweep tests were performed. In order to avoid effects on the rheological properties due to ageing, all the CNC solutions were sonicated in bath for 30 min and stirred for 10 min before use. In the stress sweep tests, a shear stress with variable amplitude τ was applied on the sample keeping the frequency f constant at 1 Hz and the temperature fixed at 25 °C.

In the frequency sweep test, a constant stress inside the linear viscoelastic regime (τ = 0.1 Pa) was applied on the sample by varying the angular frequency ω = 2πf at 25°C. The results of these two sets of measurements are reported in Figure S1, panel (a) and (b), respectively. It can be observed that the viscous modulus G" is higher than that of the elastic modulus G' for all the tested CNC solutions according to a liquid-like behaviour.

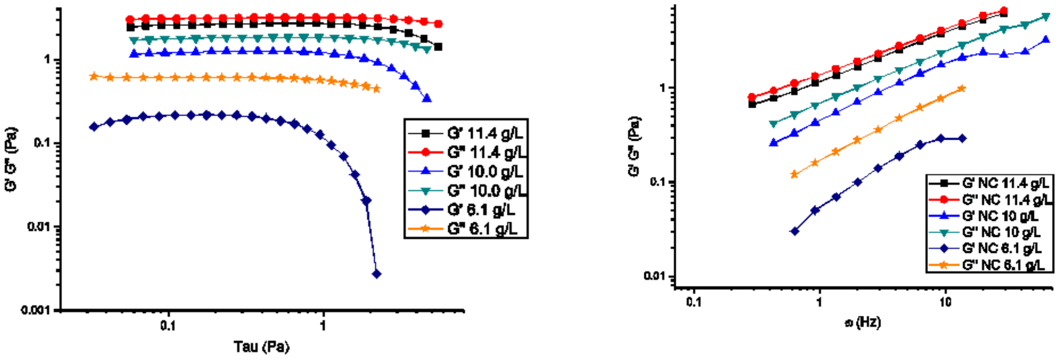

(a) (b)

Figure S1: (a) Stress sweep test. G' and G” are the elastic and the viscous modulus, respectively. τ(Pa) is the applied stress at constant frequency f = 1 Hz (T = 25 °C). (b) Frequency sweep test. $\omega = 2\pi f$ is the angular frequency of the applied stress τ= 0.1 Pa (25 °C).

Appendix B. Production of beads by pump-driven external gelation

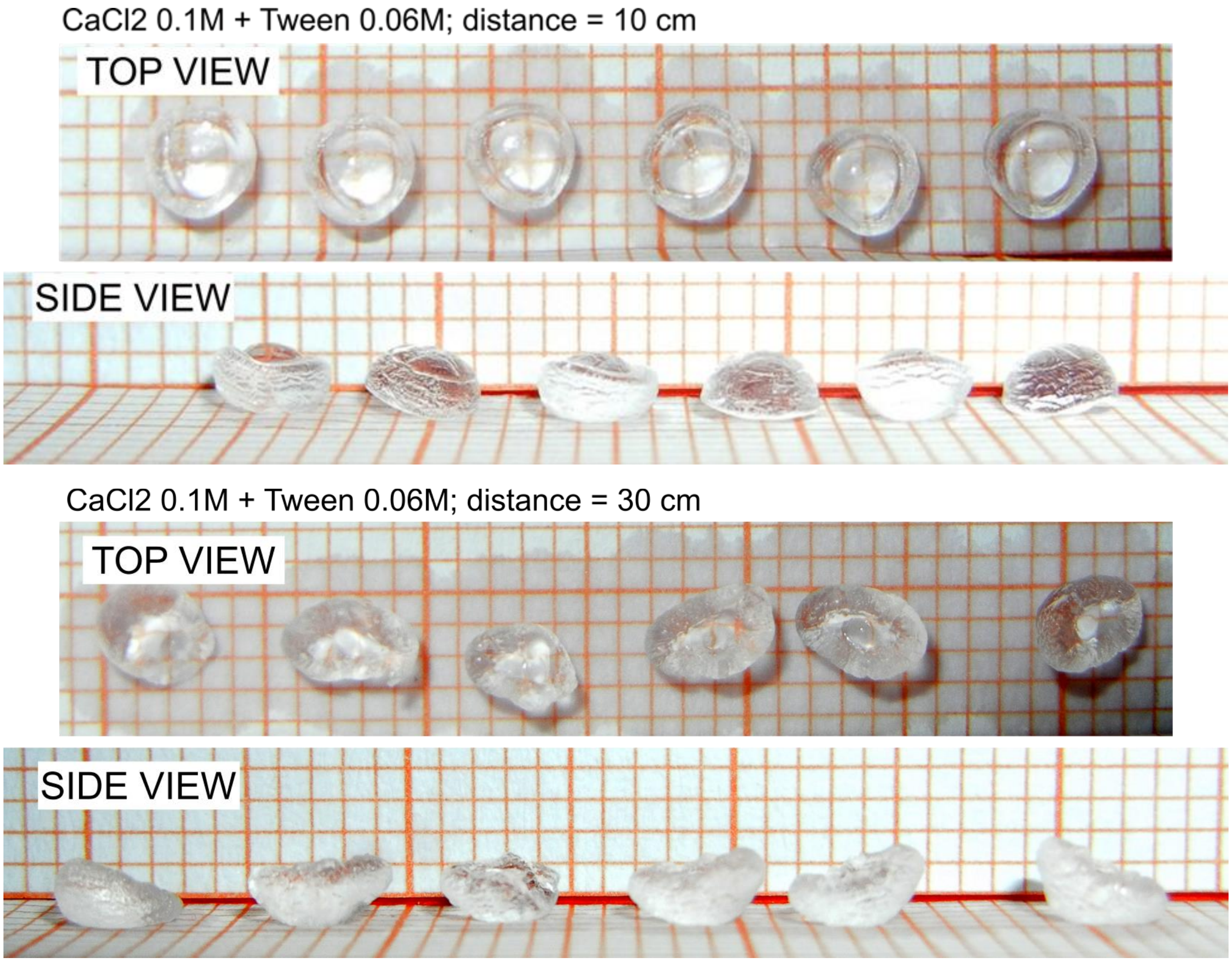

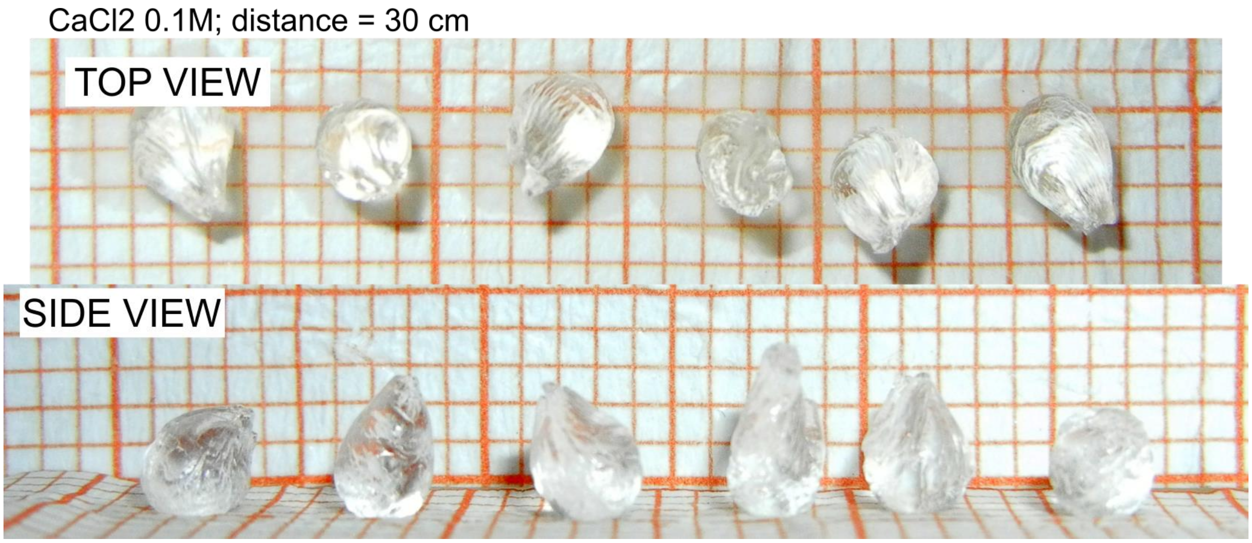

Figure S2. Representative morphologies of CNC beads produced by pump-driven external gelation. The beads were obtained by delivering a 14.7 gL^{-1}CNC solution through a needle (outer diameter d_t=636±4 μm) at a flow rate of 3.0 x10^{-3} mLs^{-1} to a reservoir which contained containing 0.1 M $CaCl_2$ and 0.06 mM Tween (rows 1-4) and 0.1 M $CaCl_2$ (rows 5-6) The distance from the gellification solution was 10 cm (rows 1-2) and 30 cm (rows 3-6). Side and top views of the beads are shown.

.

Table S1. Bead profiles at different experimental settings.

CNC(gL-1)	Collecting distance (cm)	surface tension (mNm-1)[1]	Bead shape
**8.1**	1	73	Pear-shaped
**8.1**	2	73	Pear-shaped
**8.1**	3	73	Pear-shaped
**8.1**	5	73	Pear-shaped
**8.1**	10	73	Irregular
**8.1**	20	73	Irregular
**8.1**	1	37	Pear-shaped
**8.1**	2	37	Pear-shaped
**8.1**	3	37	Concave
**8.1**	5	37	Concave
**8.1**	10	37	Concave
**8.1**	20	37	Irregular
**11,4**	1	73	Pear-shaped
**11,4**	2	73	Pear-shaped
**11,4**	3	73	Egg-shaped
**11,4**	5	73	Egg-shaped
**11,4**	10	73	Egg-shaped
**11,4**	20	73	Irregular
**11,4**	1	37	Pear-shaped
**11.4**	2	37	Pear-shaped
**11.4**	3	37	Egg-shaped
**11,4**	5	37	Concave
**11.4**	10	37	Concave
**11.4**	20	37	Concave
**14.7**	1	73	Pear-shaped
**14.7**	2	73	Pear-shaped
**14.7**	3	73	Pear-shaped
**14.7**	5	73	Concave
**14.7**	10	73	Concave
**14.7**	20	73	Quasi-spherical
**14.7**	30	73	Pear-shaped
**14.7**	1	37	Pear-shaped
**14.7**	2	37	Pear-shaped
**14.7**	3	37	Pear-shaped
**14.7**	5	37	Concave
**14.7**	10	37	Concave
**14.7**	20	37	Concave
**14.7**	30	37	Concave

[1]The surface tension value of the gelling solution in the absence (73 mNm-1) and presence (37 mNm-1) of 60 μM Tween 20. The reported shape was obtained by visual inspection of 20 beads

Appendix C. Experimental drop and bead volumes and calculation of lost and shrinkage factors

The liquid lost factor is defined as:

$$k_{LF} = \left(\frac{V_{real}}{V_{ideal}}\right)^{1/3}$$

Where V_{ideal} is the ideal drop volume which according to the Tate's 'ideal drop' definition is an approximation of the maximum drop size sustainable for any particular needle width and interfacial tension, and it gives a reasonable estimate of the actual theoretical maximum drop size [8].

A V_{ideal} value of 0.0146±0.0003 mL (herein approximated to 0.015 mL) was calculated from the theoretical Tate's law:

$$\rho g V_{ideal} = 2\pi r_t \gamma$$

where ρ is the density of the CNC solution, g is the gravitational acceleration, γ is the surface tension and r_t is the external radius of the capillary.

Where $\rho = 1.0050 \pm 0.0003$ kgL^{-1}(experimental value, see Methods section, herein approximated to 1.005 kgL^{-1}), $g = 9.813\ \mathrm{m}s^{-2}$. The γ value was set equal to the tabulated value of surface tension of dextran solution with the same concentration of CNC solution (14.7 g/L), that is $\gamma = 72\ \pm 1mN\ m^{-1}$ [17]. This value is also comparable of that of 4% CNC solution [18].

$V_{real}$was experimentally determined. A defined number of drops (200-300 drops) was produced and collected in a beaker containing water. This procedure was repeated eight times to produce a reasonable statistic.

Considering that the mass is additive, the total increment of mass due to the drops collected in the beaker was measured. Since the number of detached drops was known, by using the density value of the CNC solution, the average volume of a detached drop was calculated and set equal to V_{real}.

By using a capillary with d_t of 636±4 μm and a of CNC of 14.7 gL^{-1} a value of V_{real} of 0.0108 ±0.0002 mL was found. The error was calculated as standard deviation on the repeated measurements. No significant difference was found with CNC solutions of 10 gL^{-1}. This value was approximated to 0.011 mL for the calculations reported in this paper.

Therefore, a value of k_{LF} = 0.90 ± 0.01 was obtained.

Liquid drops can change volume upon gelation. This effect is taken into account by the shrinkage factor which is defined as:

$k_{SF} = \frac{d_p}{d_d}$, where d_p is the diameter of the Ca^{2+} crosslinked CNC beads and d_d is the volume of the bead before gelation. d_d can be calculated from the drop volume V_{real} of 0.011 mL experimentally determined (see paragraph above).

To evaluate d_p, 100 beads were produced by external gelation of a 14.7 gL^{-1} CNC solution in 0.1 M $CaCl_2$. The gelled beads were withdrawn from the solution and the surrounding excess of liquid was dried with blotting paper. Then the beads were transferred in a volume calibrated buret containing a 0.1 M $CaCl_2$ aqueous solution and their volume (V_p) was measured by recording the overall volume increment in the buret scale and dividing this value by the number of the beads.

Nine repetitions of these measurement were performed and a mean value of V_p of 0.0119±0.0007 mL was found. This value was approximated to 0.012 mL in the calculations.

From the experimental ratio:

$\frac{V_p}{V_{real}}$ a k_{SF} value of 1.1 was found. This value indicates that upon gelation step determines a slight increase in volume of the final bead with respect to the drop of the CNC solution.

Appendix D Production of beads by a centrifugal-force-driven micronozzle system

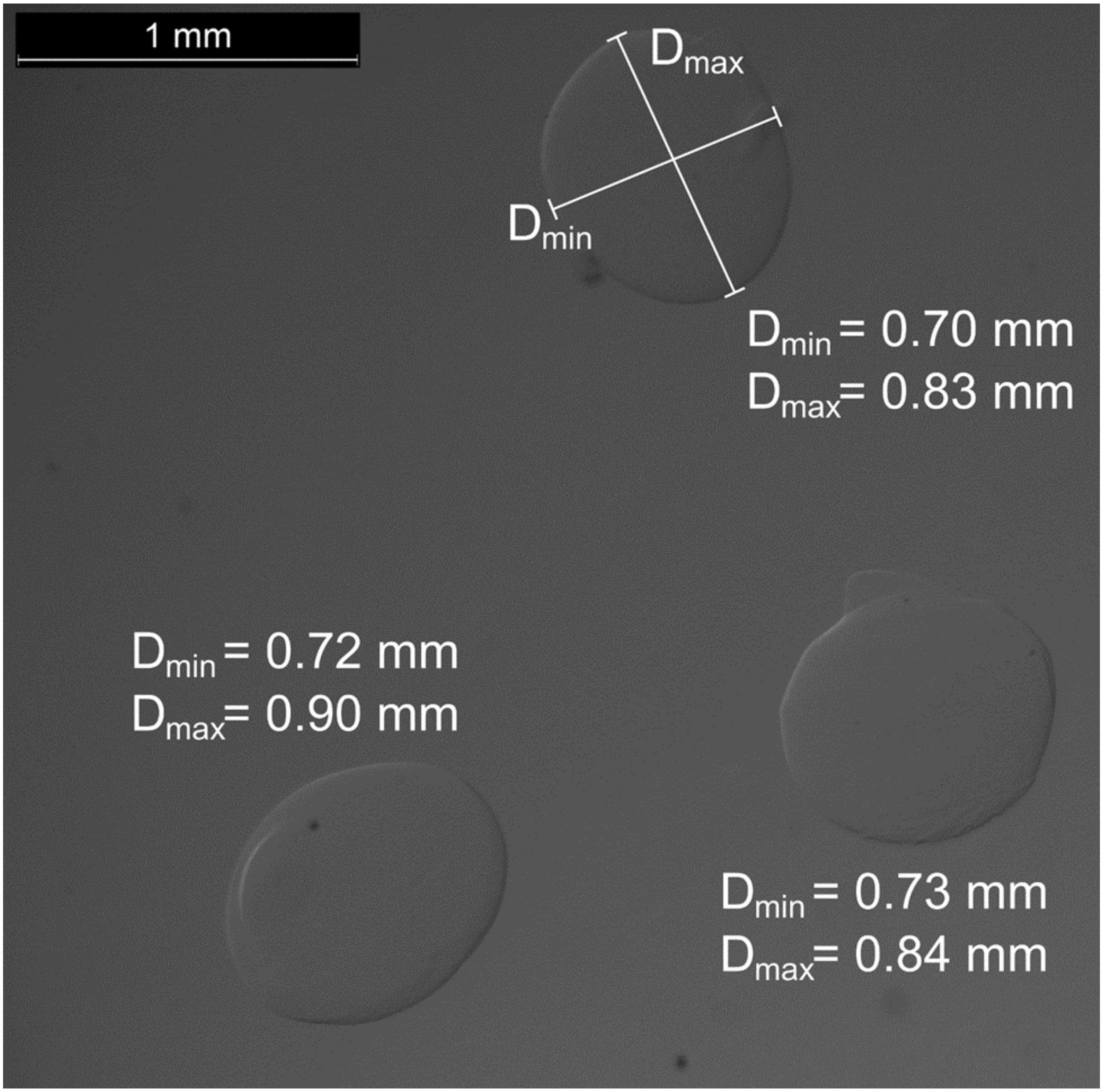

Figure S3 Microscopy images of beads obtained at different centrifuge fields. Centrifugal acceleration 823 ms^{-2}. CNC concentration 14.7 gL^{-1}, collecting distance 2 mm, external diameter of the nozzle 1240 ±10 µm.

After gelation, the beads were transferred in a Petri dish containing a 0.01 M $CaCl_2$ solution and the images were acquired by a stereo microscope (LEICA MZ16FA).

Appendix E. Morphological characterization of CNC.

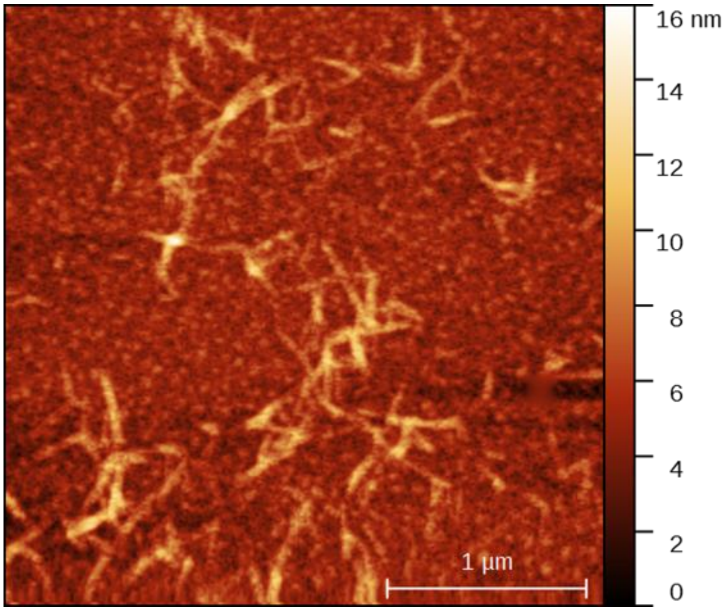

Figure S4. AFM image of CNC. CNCs were obtained by TEMPO-mediated oxidation according to the procedure of Saito et al. [24] modified by Bettotti et al. [25].

The CNC are rod-like crystalline nanostructures with 180 nm average length and 5 nm average width.
